# Exploring the association of long noncoding RNA expression profiles with intracranial aneurysms, based on sequencing and related bioinformatics analysis

**DOI:** 10.1186/s12920-020-00805-x

**Published:** 2020-10-06

**Authors:** Yi Sun, Yeying Wen, Qishuang Ruan, Le Yang, Shuna Huang, Xingyan Xu, Yingying Cai, Huangyuan Li, Siying Wu

**Affiliations:** 1 Department of Epidemiology and Health Statistics, School of Public Health, Fujian Medical University, Minhou County, Fuzhou, 350122 China; 2 Department of Orthopedics, Fujian Medical University Union Hospital, Fuzhou, 350001 China; 3 Department of Preventive Medicine, School of Public Health, Fujian Medical University, Minhou County, Fuzhou, 350122 China

**Keywords:** RNA sequencing, Expression profiles, Bioinformatics analysis, Predictive value, Intracranial aneurysms

## Abstract

**Background:**

The present study aims to investigate the complete long non-coding RNA (lncRNA) and messenger RNA (mRNA) expression profiles in Intracranial aneurysm (IA) patients and controls by RNA sequencing, which reveals the lncRNA with predictive value for IA risk.

**Methods:**

The comprehensive lncRNA and mRNA expression profiles were detected by RNA-Seq in human IA walls and superficial temporal arteries (STAs), followed by bioinformatics analyses, such as GO analysis, KEGG pathway analysis, and CNC network construction. Subsequently, qRT-PCR was used to profile the expression levels of selected lncRNA (lncRNA ENST000000576153, lncRNA ENST00000607042, lncRNA ENST00000471220, lncRNA ENST00000478738, lncRNA MALAT1, lncRNA ENST00000508090 and lncRNA ENST00000579688) in 30 (small) or 130 (large) peripheral blood leukocytes, respectively. Multivariate logistic regression was utilized to analyze the effects of lncRNA on IA. Receiver operating characteristic (ROC) curve was further drawn to explore the value of lncRNA in predicting IA.

**Results:**

Totally 900 up-regulated and 293 down-regulated lncRNAs, as well as 1297 up-regulated and 831 down-regulated mRNAs were discovered in sequencing. Enrichment analyses revealed that they were actively involved in immune/inflammatory response and cell adhesion/extracellular matrix. Co-expression analysis and further enrichment analyses showed that five candidate lncRNAs might participate in IA’s inflammatory response. Besides, after controlling other conventional risk factors, multivariate logistic regression analysis disclosed that low expression of lncRNA ENST00000607042, lncRNA ENST00000471220, lncRNA ENST00000478738, lncRNA MALAT1 in peripheral blood leukocytes were independent risk factors for IA. LncRNA ENST00000607042 has superior diagnostic value for IA.

**Conclusions:**

This study reveals the complete lncRNAs expression profiles in IA. The inflammatory response was closely related to IA. Besides, lncRNA ENST00000607042 might be a novel biomarker for IA risk.

## Background

Intracranial aneurysm (IA) is a localized lesion of the cerebral arteries that affect 2 to 3% of the general population [1]. IA rupture can cause aneurysmal subarachnoid hemorrhage (aSAH), which leads to a mortality rate of 30–40% and poor postoperative recovery of patients who survive it [2–4]. Researchers have identified several risk factors for IA’s development, including family history of IA, smoking, alcohol, family history of hypertension, older age and female [5–7]. But the genetic pathology of IA remains unclear.

Long non-coding RNAs (lncRNAs) are non-coding RNAs that are _> 200 nucleotides in length_ and widely distributed in the nucleus and cytoplasm, especially the nucleus [8]. Multiple studies have revealed that lncRNA can be involved in the inflammatory process, vascular smooth muscle cells (SMCs) phenotypic transition, endothelial cell function regulation and lipid metabolism [9–12], which play essential roles in IA. Therefore, these results indicate that lncRNA may be associated with the pathogenesis of IA.

Fewer studies investigate the relationship between lncRNA and IA [13, 14]. In this study, we explored the differential expression of lncRNAs and messenger RNAs (mRNAs) between IA patients and controls based on sequencing technology. The results indicated that several lncRNAs were closely involved in the pathogenesis of IA. Five candidate lncRNA were further validated by subsequent large-sample peripheral blood PCR experiments in IA and healthy controls. Association and predictive value of lncRNAs and IA were evaluated using receiver operating characteristic curve (ROC) and multivariate logistic regression analysis. In summary, the study aims to reveal the complete lncRNA expression profiles in IA, and to further explore the predictive value of lncRNA for IA.

## Methods

### Patients and samples selection for RNA sequencing

**Fig. 1:**
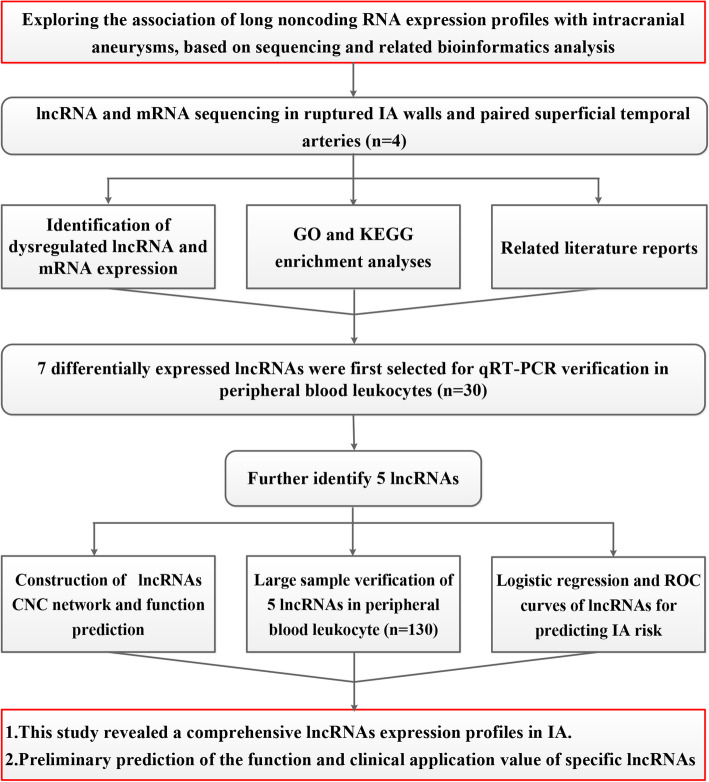
Illustrative figure of the study

A total of 4 ruptured IA walls (one is an anterior communicating artery aneurysm, and the other three are middle cerebral artery aneurysms) and 4 paired superficial temporal arteries were collected from patients at the Second Affiliated Hospital of Fujian Medical University between December 2017 to March 2018. Their demographic characteristics are shown in supplementary Table 1 (Table S1), Fig. 1 is the illustrative figure of the study. The peripheral blood of the above patients was also collected. Patients were eligible when: 1. Diagnosis of ruptured IA (by CTA, MRA or DSA). 2. Age older than 18 years. 3. No major diseases such as malignant tumors and arteriovenous malformations. 4. Patient data is complete. The exclusion criteria were as follows: 1. The cause of subarachnoid hemorrhage is unknown. 2. Malignant tumor. 3. Other cerebral arteriovenous malformations or cerebral arteriovenous fistulas. 4. Aneurysms caused by trauma or other factors. The corresponding control participants were diagnosed as free of IA who were attending a regular health check-up, including clinical examinations (CTA, MRA or DSA) and medical history assessments. Our research protocol meets the Helsinki declaration and the plan was approved by the Fujian Medical University Ethics Committee.

### Study population for exploration

Totally 300 participants (150 cases and 150 controls) were enrolled and confirmed their participation by signing the consent forms based on inclusion and exclusion criteria. After corresponding the gender and age by propensity score matching (matching tolerance = 0), 130 patients and 130 controls were enrolled finally, including the blood sample. Head CTA, MRA or DSA were used to diagnosed or excluded IA. Comprehensive data were collected including age, gender, marital status, smoking, alcohol drinking, family history of stroke, tea-drinking, diets, physical exercise, SBP, DBP, TG. Alcohol drinkers and Tea drinking were defined the same as the literature [15, 16]. Active exercise participants were the individuals who exercised at least 20 min once a week. Individuals who consumed at least 20 packs of cigarettes or smoked one cigarette/day for at least 1 year were defined as smokers [17].

### RNA extraction

First, peripheral blood leukocytes were isolated from 130 cases and control blood samples. Then, TRIzol reagent (Takala, USA) were used to extracted total RNA according to the kit’s instructions. RNA samples’ quality and integrity were determined. Samples with an OD_260_/OD_280_ ratio between 1.8–2.0 were accepted.

### RNA sequencing

Total RNA was ribo-consumed by Ribo-ZeroTM Removal Kit (Epicentre, USA) and then treated with RNase (20 U/L) at 37 °C for an hour. Finally, RNA Cleanup magnetic beads (Geneaid, USA) was used to purify. Illumina HiSeq 4000 (Illumina, Inc.) was used for sequencing according to the manufacturer’s protocol. FastQC (v0.11.3) (The Babraham Institute, Cambridge, UK) was used to quality control. Libraries were constructed according to the standard TruSeq protocol. A fluorometer (Qubit 4.0) was then used to evaluate the database centralization while detecting its quantity by Agilent bioanalyzer 2100. The sequencing experiment’s veracity quantification was identified by Library Quantification Kit (Kapa Biosystems, USA). Subsequently, data analyses were performed in silico. At least 1.5 folds with *P* < 0.05 was considered a significant difference..

### GO and KEGG pathway analysis

DAVID was used for GO analysis (http://www.geneongoloty.org/) and KEGG pathway analysis (http://www.genome.jp/kegg/). The meaningful annotations of genes and gene products were constructed by GO analysis. KEGG pathway analysis was used to capture the clusters of pathways involved in the molecular interaction and reaction networks. The log10 (*p*-value) denotes the enrichment score.

### Construction of the CNC network

LncRNA and mRNA with Pearson correlation> 0.90 or < − 0.90, *P* < 0.05 were selected to draw the network using Cytoscape software [18]. Concentration is treated as the number of nodes associated with other nodes in the network analysis. The degree can determine the relative importance of a network [19].

### qRT-PCR assay

PrimeScript RT Reagent Kit (Takara Bio Inc., Shiga, Japan) was performed to reverse transcription of quantified RNA based on the manufacturer’s instructions. The SYBR® Premix Ex Taq™ II kit (Takara Bio Inc., Shiga, Japan) was used for qRT-PCR and performed on the LightCycler 480 Real-Time PCR System (Roche, Switzerland). The 2^-ΔΔCt^ method was used to represent the expression level of lncRNA. The internal control was β-actin. Supplementary table 2 (Table S2) listed the primer sequences.

### Statistical analysis

Chi-square (χ^2^) test and t-test were used to analyze qualitative and quantitative data respectively. Multivariate analysis was followed. Pearson coefficient analysis was utilized to assess the co-expression network. ROC curve analysis was conducted to determine the value of lncRNA. Analyses were accomplished by using SPSS 19.0. A two-tailed *P* < 0.05 was considered significant.

## Results

### LncRNA and mRNA expression profiles

**Fig. 2:**
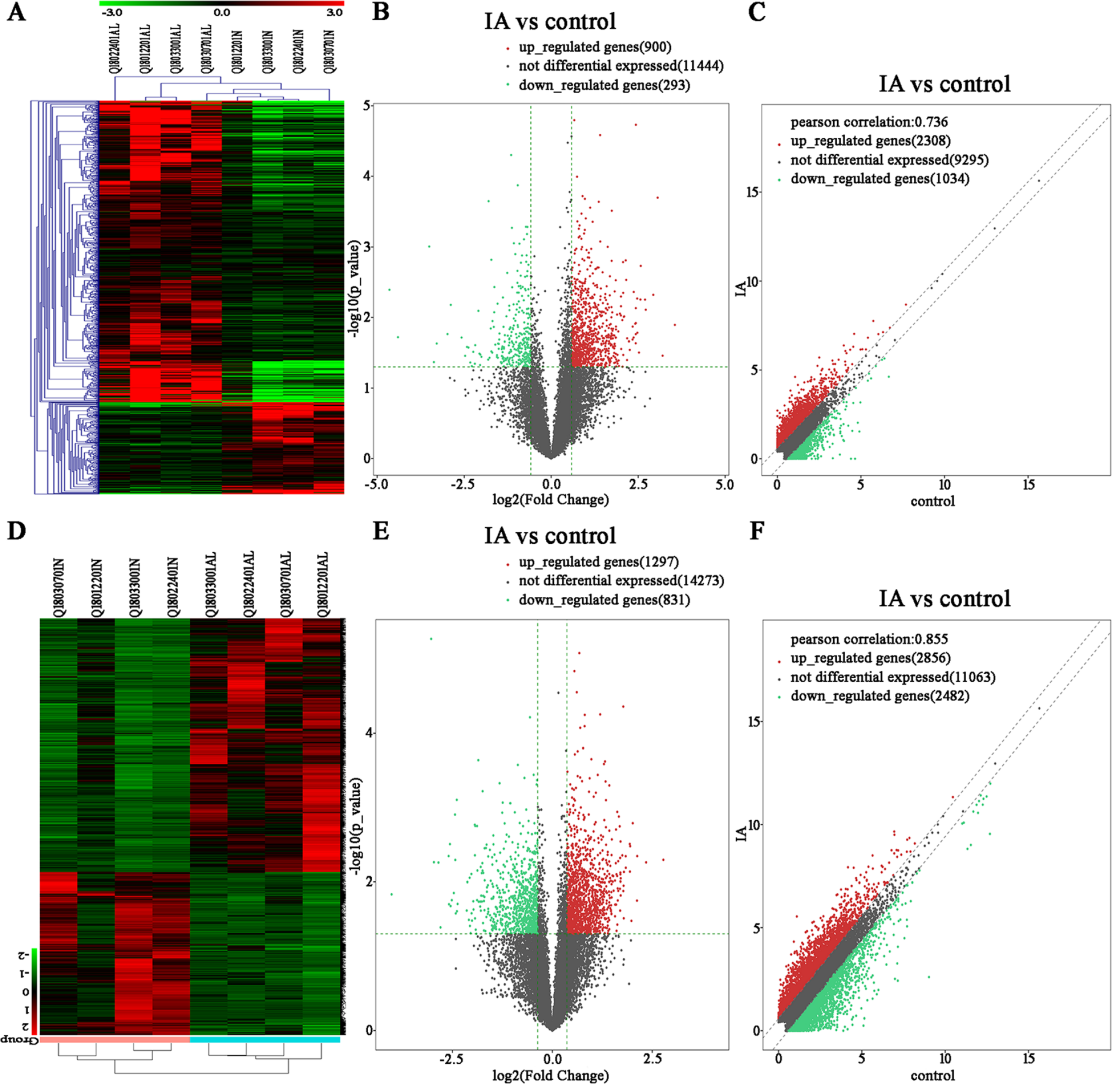
LncRNA and mRNA expression profiles. **a**, **d** Heatmap analysis. **b**, **c** Volcano plot and scatter plot foe lncRNA. **e**, **f** Volcano plot and scatter plot for mRNA. Red indicates relatively up-regulated RNAs, green represents relatively down-regulated RNAs, whereas black indicates RNAs with no significant difference

The heatmap analysis showed that lncRNA and mRNA profiles were able to differentiate IA patients from controls (Fig. 2a,d). A total of 3342 lncRNAs were significant difference expression (FC ≥ 1.5), of which 2308 lncRNAs transcripts were up-regulated and 1034 lncRNAs were down-regulated (Fig. 2b). Further screening for statistically significant lncRNAs. 1193 lncRNAs were significantly different (FC ≥ 1.5, *P* < 0.05), among which 900 lncRNAs were up-regulated and 293 lncRNAs were down-regulated (Fig. 2c). And the top 10 up-regulated and top 10 down-regulated lncRNAs were displayed in Table 1. As to mRNA, totally 5338 differentially expressed mRNAs were identified (FC ≥ 1.5), in which 2856 mRNAs were obviously up-regulated while 2482 mRNAs were obviously down-regulated in IA patients compared with controls (Fig. 2e). Statistically significant mRNAs were further screened. Including 1297 up-regulated mRNA and 831 down-regulated mRNA (FC ≥ 1.5, *P* < 0.05), (Fig. 2f).

**Table 1:** Top 10 upregulated and 10 downregulated lncRNAs

lncRNA ID	Location	FC (abs)	*P*	Trend
LncRNA ENST00000508090	chr5	11.7467	0.0128	Up
LncRNA ENST00000576153	chr17	9.2067	0.0347	Up
LncRNA ENST00000569478	chr16	8.3411	0.0002	Up
LncRNA ENST00000478738	chr2	6.5808	0.0078	Up
LncRNA ENST00000463972	chr6	6.4857	0.0056	Up
LncRNA ENST00000607042	chr14	5.9560	0.0019	Up
LncRNA ENST00000471220	chr1	5.9160	0.0056	Up
LncRNA ENST00000492361	chr1	5.7511	0.0313	Up
LncRNA ENST00000466288	chr2	5.5660	0.0165	Up
LncRNA ENST00000583222	chr17	5.5460	0.0054	Up
LncRNA ENST00000446406	chr11	25.0729	0.0041	Down
LncRNA ENST00000469162	chr1	21.1478	0.0191	Down
LncRNA ENST00000469162	chr1	21.1478	0.0191	Down
LncRNA ENST00000579688	chr17	11.3504	0.0010	Down
LncRNA ENST00000474353	chr10	10.3412	0.0427	Down
LncRNA ENST00000487727	chr9	9.7713	0.0233	Down
LncRNA ENST00000483064	chr10	7.8829	0.0172	Down
LncRNA ENST00000412788	chr11	7.3864	0.0066	Down
LncRNA ENST00000374673	chr1	5.9622	0.0193	Down
LncRNA ENST00000532150	chr11	5.5509	0.0348	Down

### GO and KEGG pathway analyses

**Fig. 3:**
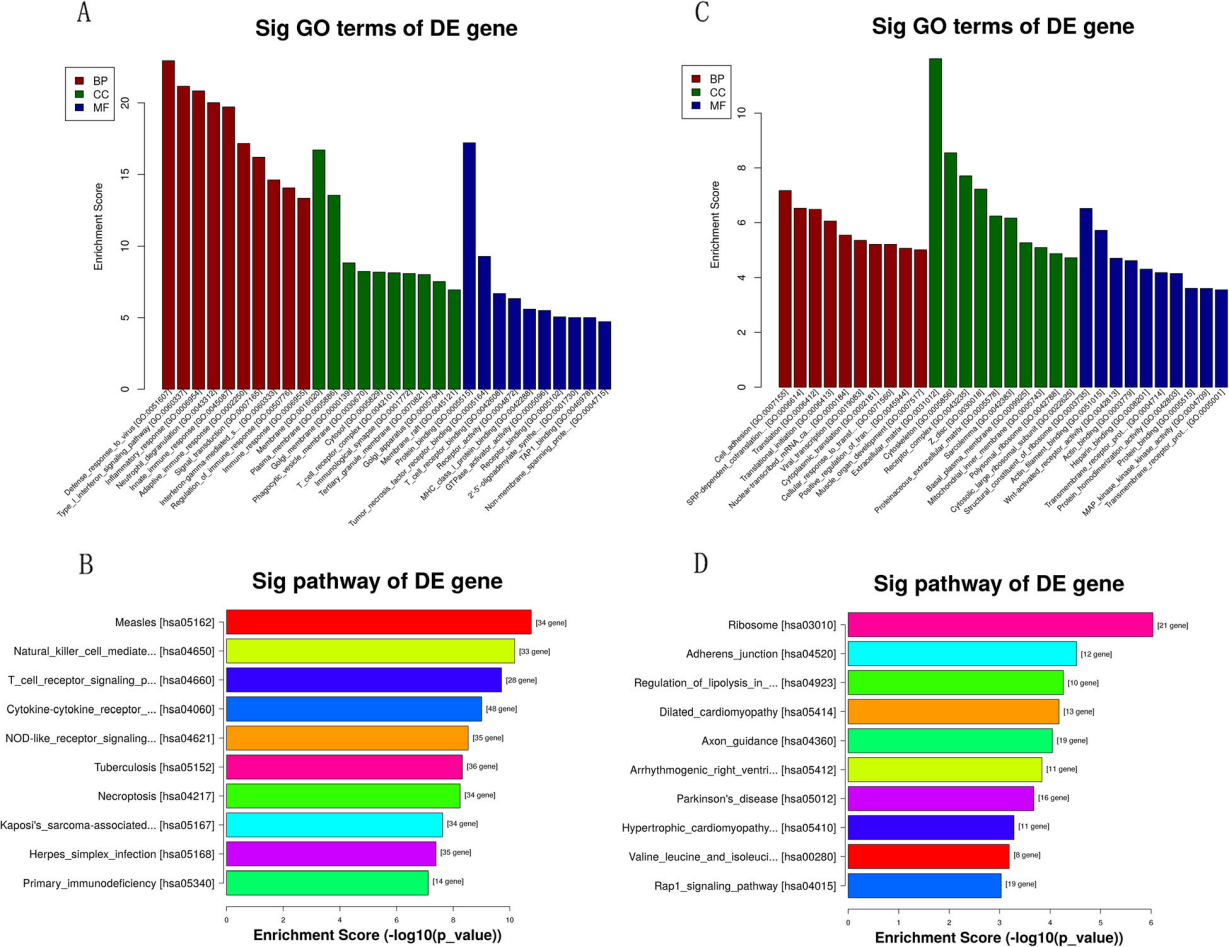
GO and KEGG enrichment analyses. **a**, **b** GO and KEGG enrichment analyses of up-regulated genes; **c**, **d** GO and KEGG enrichment analyses of down-regulated genes

As for the up-regulated target genes, GO analysis showed that immune/inflammatory response were most of the functional terms. KEGG pathway analyses revealed that T cell receptor signaling pathway was one of the most enriched pathway (Fig. 3a-b). As for the down-regulated target genes, The most of the functional terms were related to cell adhesion and extracellular matrix. The genes were most enriched in adherens junction and lipolysis regulation in adipocytes (Fig. 3c-d).

### Verify the accuracy of sequencing and small sample qRT-PCR preliminary screening

**Fig. 4:**
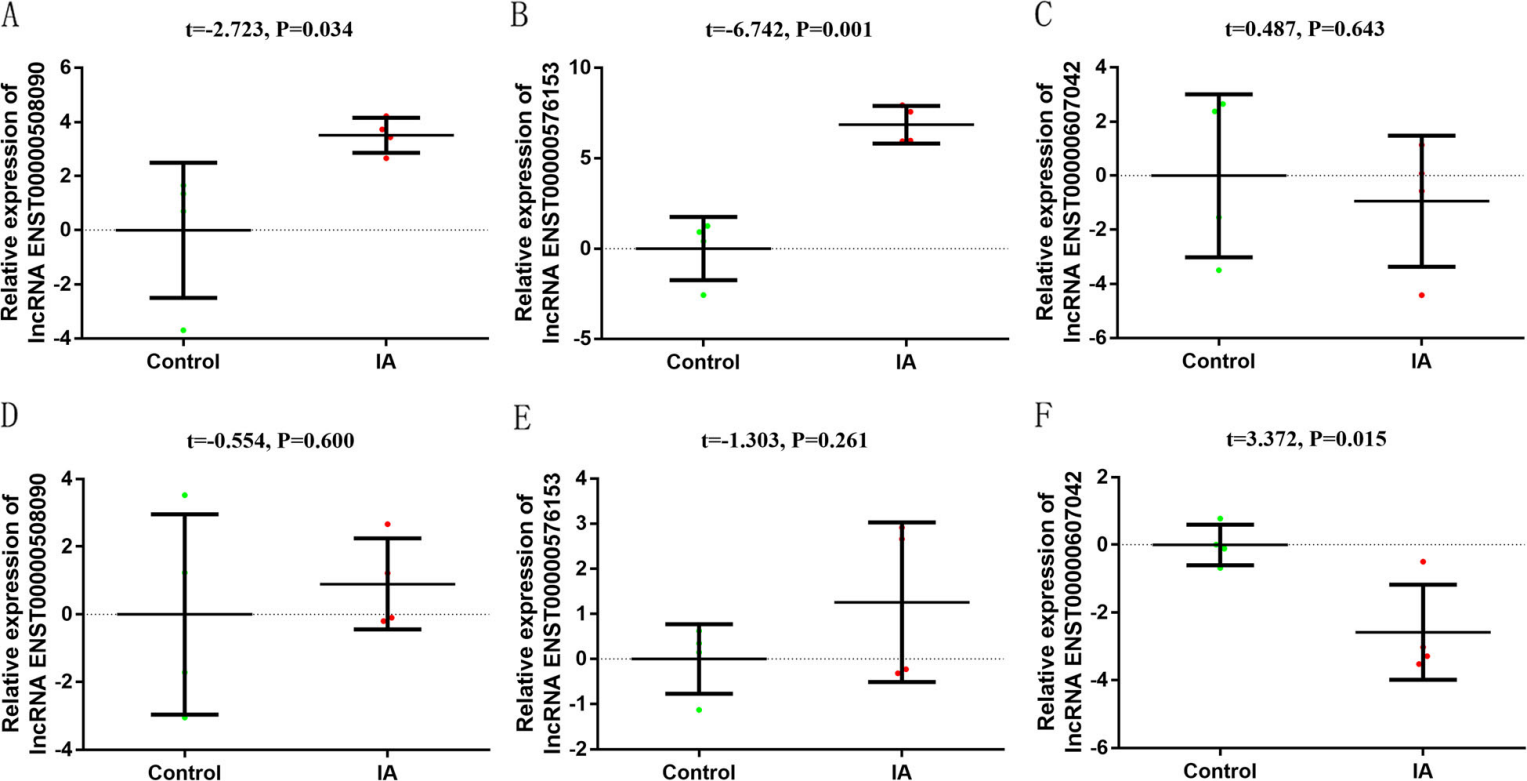
LncRNAs expression level in different samples. **a**-**c** LncRNA expression level in tissues; **d**-**f** LncRNAs expression level in peripheral blood leukocytes. (2^-ΔΔCt^ was used to describe the expression of lncRNA. After log 2 logarithmic transformation, t test was used to compare the difference of lncRNA between the two groups)

Based on the lncRNA fold change, *p*-values and related bioinformatics analysis, three lncRNAs were selected to confirm the sequencing data (lncRNA ENST00000508090, lncRNA ENST00000607042 and lncRNA ENST000000576153). As shown in Fig. 4a-c, lncRNA ENST00000508090 and lncRNA ENST000000576153 expression in tissues were consistent with the RNA-seq results and the expression level of lncRNA ENST00000607042 in tissues was not statistically significant between the two groups. All these results confirmed the accuracy of sequencing. We also observed the expression of the above three lncRNAs in the same human peripheral blood leukocytes. As shown in Fig. 4d-f, lncRNA ENST00000607042, lncRNA ENST00000508090 and lncRNA ENST000000576153 showed the same expression trend in IA tissues and peripheral blood leukocytes.

**Fig. 5:**
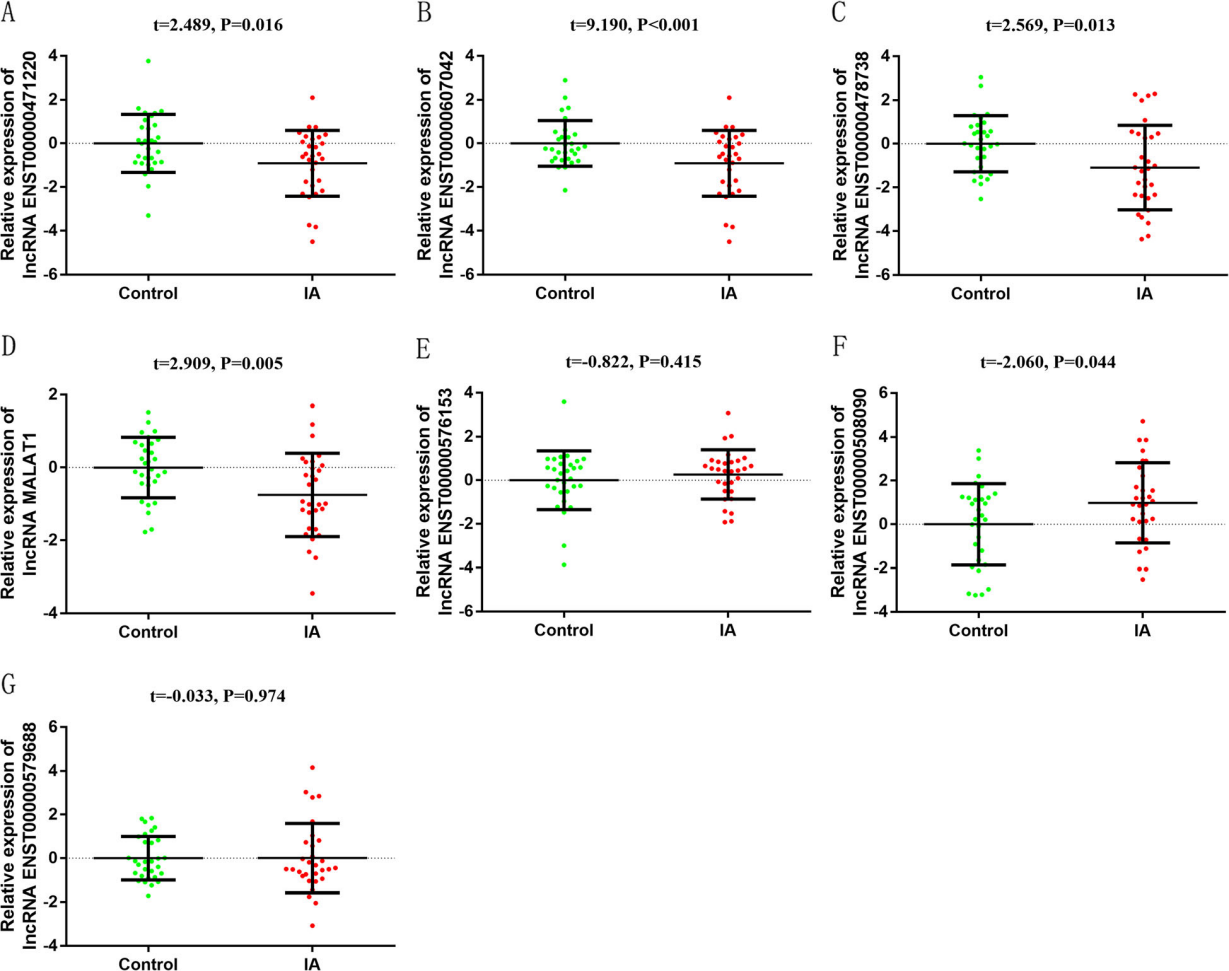
Expression level of selected lncRNAs in 30 cases and controls. **a** lncRNA ENST00000471220; **b** lncRNA ENST00000607042; **c** lncRNA ENST00000478738; **d** lncRNA MALAT1; **e** lncRNA ENST000000576153; **f** lncRNA ENST00000508090; **g** lncRNA ENST00000579688. (2^-ΔΔCt^ was used to describe the expression of lncRNA. After log 2 logarithmic transformation, t test was used to compare the difference of lncRNA between the two groups)

Next, a total of seven differentially expressed lncRNAs (lncRNA ENST000000576153, lncRNA ENST00000607042, lncRNA ENST00000471220 lncRNA ENST00000478738, lncRNA MALAT1, lncRNA ENST00000508090 and lncRNA ENST00000579688) were further selected using the same screening criteria for qRT-PCR verification among 30 cases and controls matched for age and gender. The expression level of lncRNA ENST00000607042, lncRNA ENST00000471220 and lncRNA ENST00000478738, lncRNA ENST00000508090 and lncRNA MALAT1 in peripheral blood were significantly different between the two groups (Fig. 5). Considering that the CT value of ENST00000508090 in peripheral blood leukocytes is too large, the expression is relatively low, so we did not continue to select this lncRNA for further research. Finally, we selected five lncRNAs with larger differential expression and better lncRNA expression levels for subsequent research, namely lncRNA ENST00000471220, lncRNA ENST00000607042, lncRNA ENST00000478738, MALAT1, lncRNA ENST000000576153.

### Construction of five differential expressed lncRNAs CNC network and function prediction

**Fig. 6:**
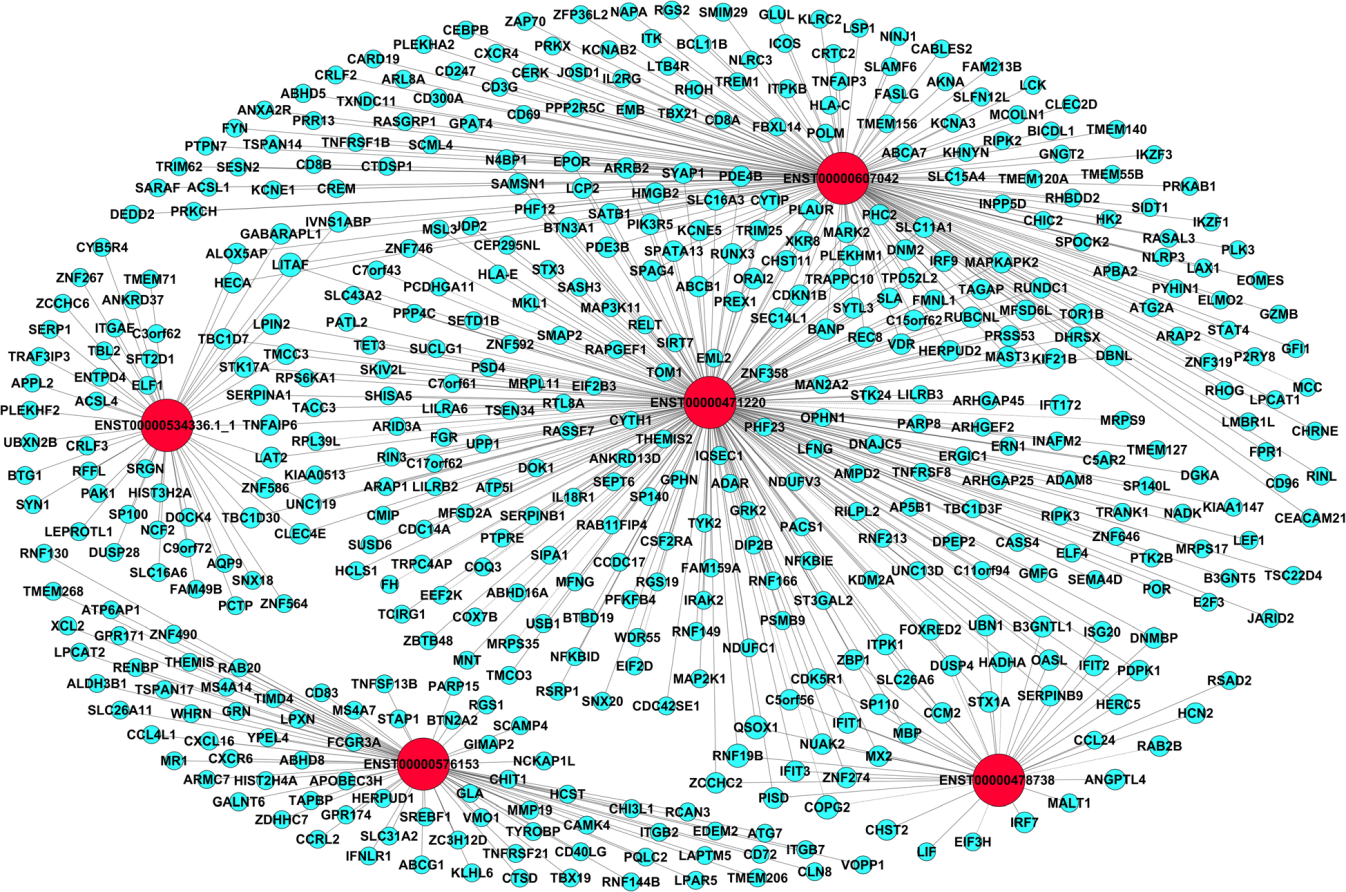
Network of lncRNAs-mRNAs. The red nodes represent lncRNAs, and the blue nodes represent mRNAs. The dotted line indicates a negative correlation and the solid line indicates a positive correlation

**Fig. 7:**
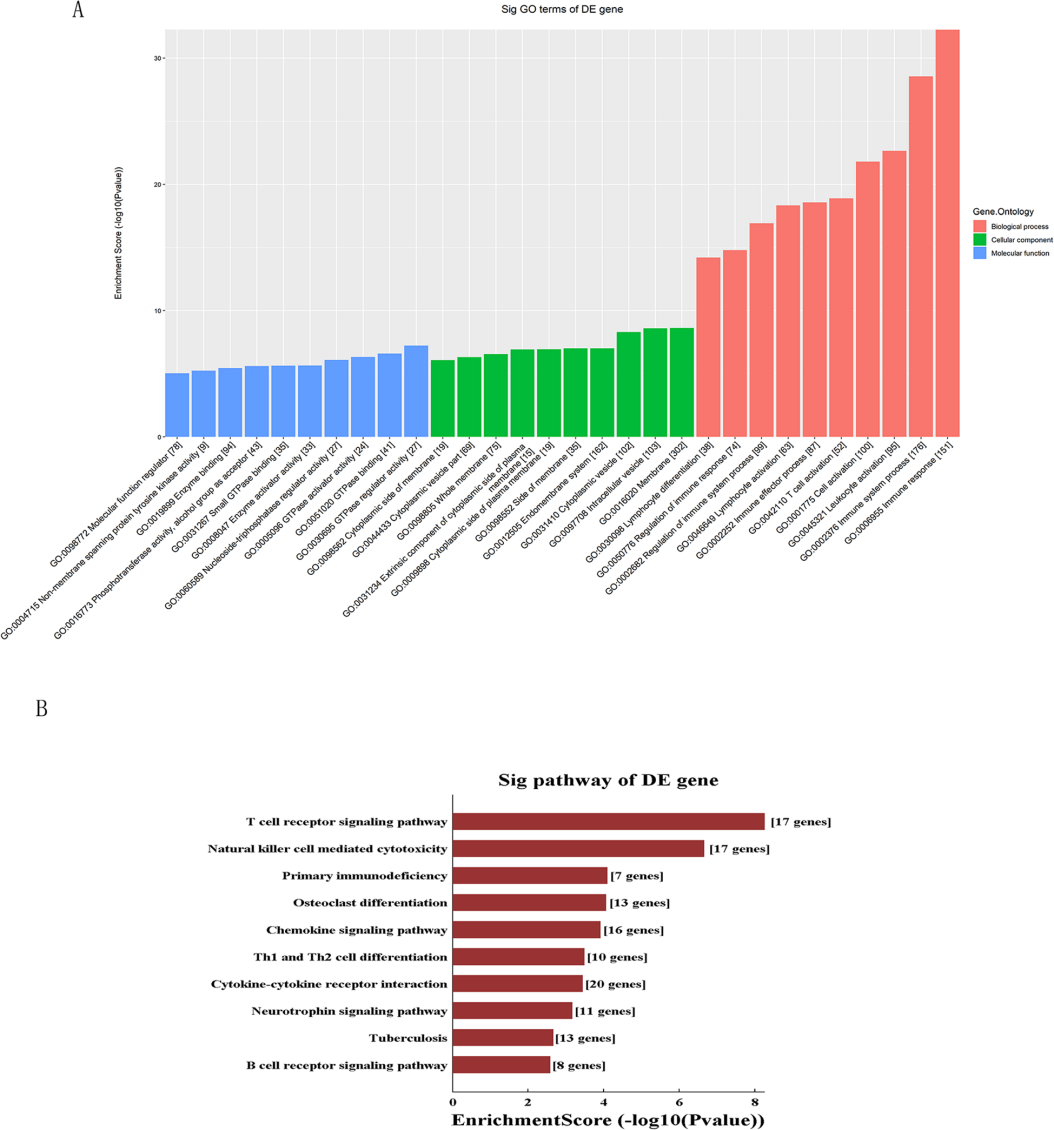
Enrichment analyses for the co-expression genes of the 5 candidate lncRNAs. **a** GO analyses; **b** KEGG enrichment analyses

To explore the potential interplay between lncRNAs and mRNAs, We next constructed the gene CNC network between candidate lncRNAs and differential expression mRNAs based on Pearson correlation> 0.90 or < − 0.90 and *P* < 0.05. As shown in Fig. 6, five lncRNAs and 593 mRNAs were included in this network, suggesting that a significant correlation exists between the expression profile of lncRNAs and mRNAs. Interestingly, we observed many mRNAs that may be involved in the pathogenesis of IA. Such as Eukaryotic elongation factor 2 kinase (EEF2K) [20], Scm polycomb group protein-like 4 (SCML4) [21], Matrix metalloproteinases 19 (MMP19) [22], Lymphoid enhancer factor 1 (LEF1) [23]. The further GO analyses revealed that the co-expression genes of the 5 candidate lncRNAs were related to T cell activation and leukocyte activation, etc. And KEGG enrichment analyses indicated that the genes were most enriched in the T cell receptor signaling pathway, cytokines-cytokines receptor interaction and chemokine signaling pathway. These items and pathways are closely related to the development of IA (Fig. 7).

### Large sample verification of candidate lncRNAs in peripheral blood leukocyte

**Fig. 8:**
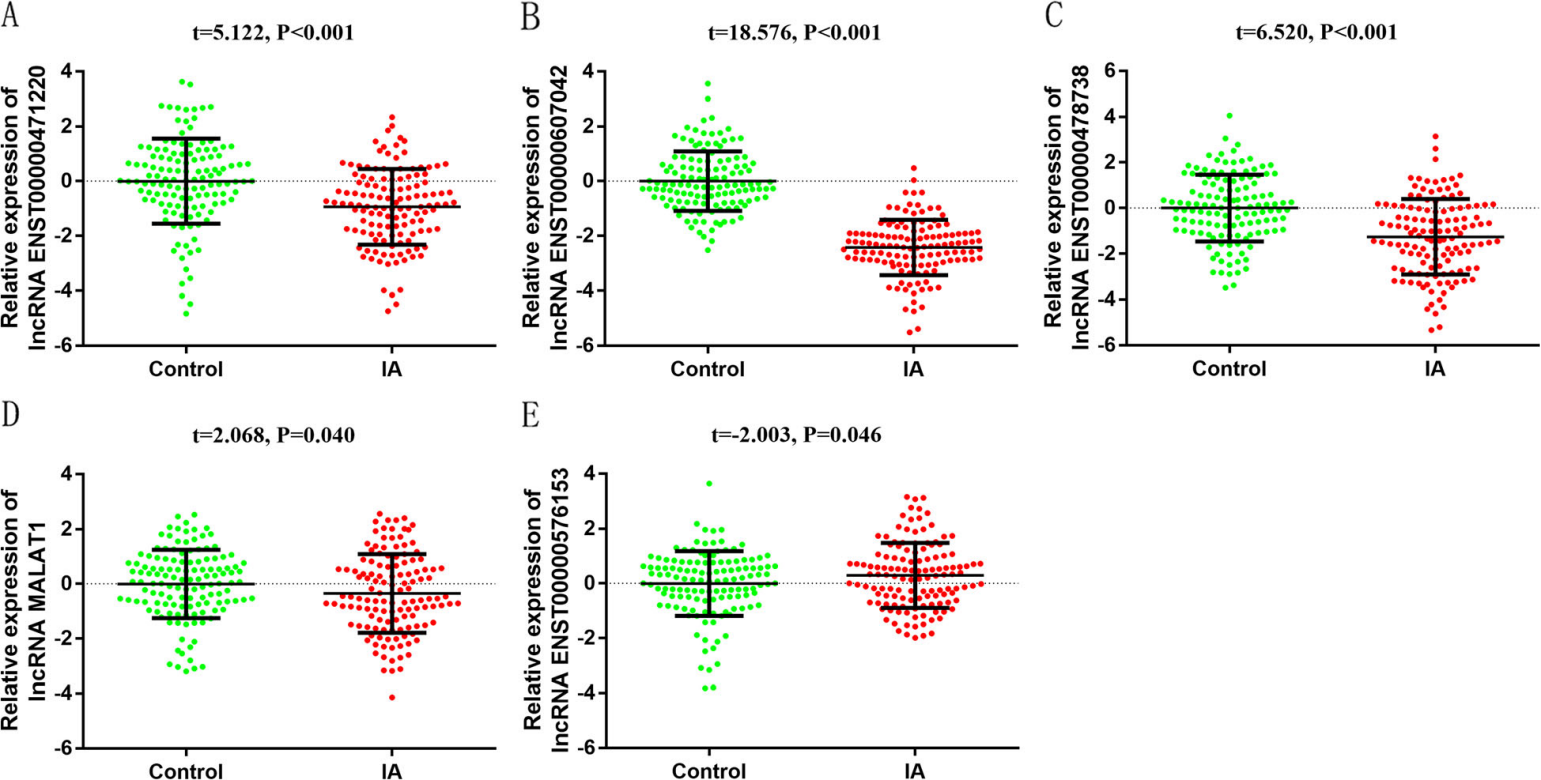
Expression level of selected lncRNAs in 130 cases and controls. **a** lncRNA ENST00000471220; **b** lncRNA ENST00000607042; **c** lncRNA ENST00000478738; **d** lncRNA MALAT1; **e** lncRNA ENST000000576153 (2^-ΔΔCt^ was used to describe the expression of lncRNA. After log 2 logarithmic transformation, t test was used to compare the difference of lncRNA between the two groups)

A total of 130 IA patients and healthy people were collected for lncRNA qRT-PCR validation. The characteristics of 130 IA patients and controls were listed in Table 2. No significant difference was found in age, gender, marital status (all *P* > 0.05). People who smoke, high-salty diet were highly prone to IA. However, tea-drinking and physical exercise were protected factors for IA, consistent with current research results [24–28]. The expression level of lncRNA ENST00000607042, lncRNA ENST00000471220 and lncRNA ENST00000478738, lncRNA ENST00000576153 and lncRNA MALAT1 were significantly different between the 2 groups (Fig. 8a-e). The expression trend of lncRNA ENST00000607042, lncRNA ENST00000471220, lncRNA ENST00000478738 in blood differed from that in sequencing.

**Table 2:** Demographic characteristics of IA patients and controls in qRT-PCR

Characteristics	Controls (*n =* 130)	IAs (*n =* 130)	χ^2^	*P* Value
Age			0	1.000
< 60	105 (80.8)	105 (80.8)		
≥ 60	25 (19.2)	25 (19.2)		
Gender			0	1.000
Male	59 (45.4)	59 (45.4)		
Female	71 (54.6)	71 (54.6)		
Marital status			2.149	0.143
Marriage	124 (95.4)	118 (90.8)		
Single and others	6 (4.6)	12 (9.2)		
Smoking			30.806	< 0.001
No	123 (94.6)	88 (67.7)		
Yes	7 (5.4)	42 (32.3)		
Alcohol			3.594	0.059
No	70 (53.8)	85 (65.4)		
Yes	60 (46.2)	45 (34.6)		
High-salt diets			17.732	< 0.001
No	107 (82.3)	76 (58.5)		
Yes	23 (17.7)	54 (41.5)		
Tea drinking			32.319	< 0.001
No	21 (16.2)	76 (49.2)		
Yes	109 (83.8)	54 (50.8)		
Physical exercise			29.365	< 0.001
No	32 (24.6)	75 (57.7)		
Yes	98 (75.4)	55 (42.3)		
Family history of stroke				0.684^a^
No	128 (98.5)	126 (96.9)		
Yes	2 (1.5)	4 (3.1)		

^a^Fisher ‘s exact test

### ROC curves of related lncRNA for predicting IA risk

**Fig. 9:**
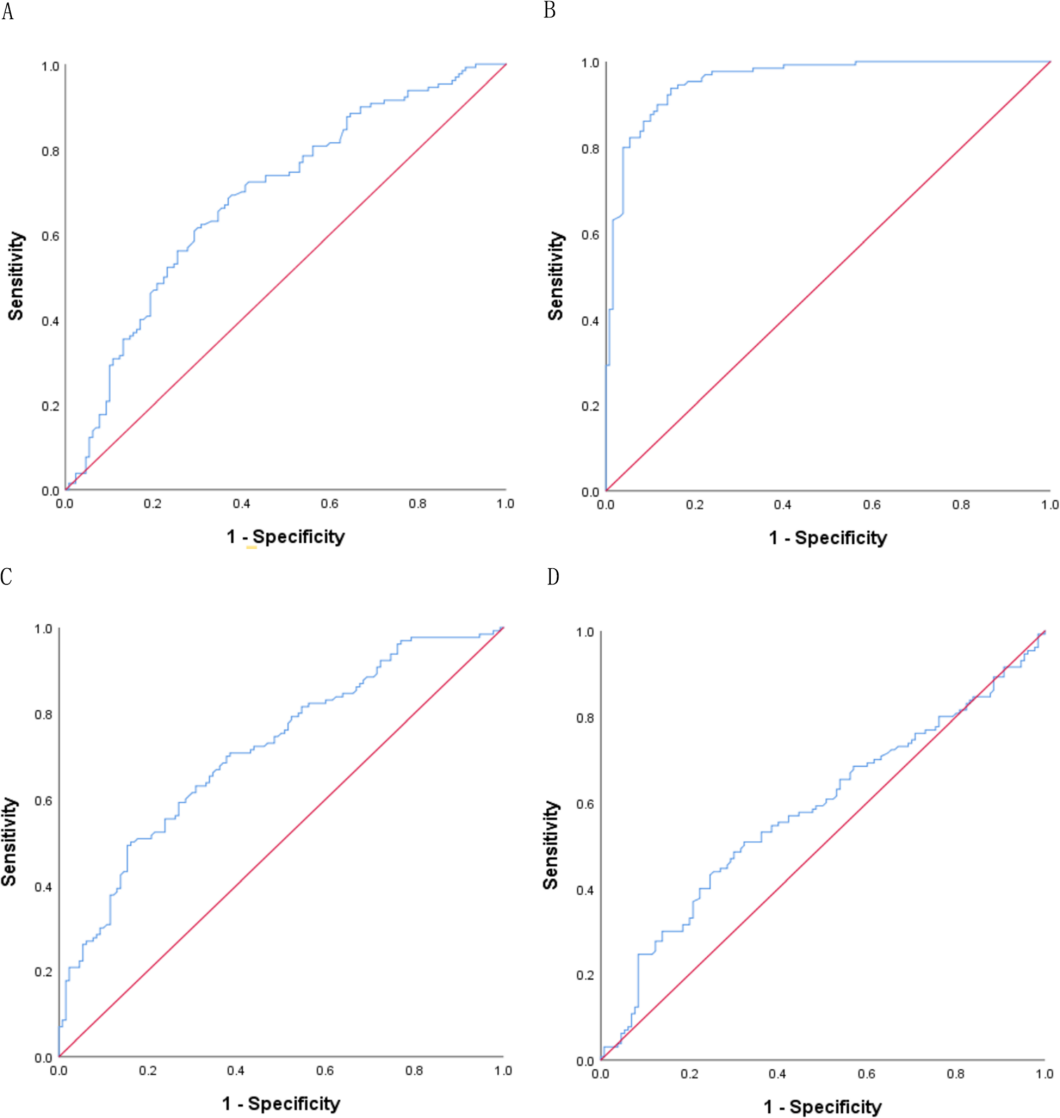
The ROC curve of lncRNAs. **a** lncRNA ENST00000471220; **b** ENST00000607042; **c** ENST00000478738; **d** MALAT1

We further performed a logistic regression analysis to predict the risk of IA. General demographic characteristics (gender (0 = man,1 = woman), age (0 = < 60, 1 = ≥60), marital status (0 = Marriage,1 = Single and others)) and variables with significant differences in univariate analysis (smoking(0 = no,1 = yes), tea drinking (0 = no,1 = yes), physical exercise (0 = no,1 = yes) were included in the logistic model (Enter). The results disclosed that low expression of lncRNA ENST00000471220 (0R = 0.604, 95%CI: 0.482–0.756), lncRNA ENST00000607042 (0R = 0.077, 95%CI: 0.037–0.160), lncRNA ENST00000478738 (0R = 0.616, 95%CI: 0.497–0.764) and lncRNA MALAT1 (0R = 0.754, 95%CI: 0.597–0.951) were risk factors for IA (Table 3). ROC curves showed that the AUC of lncRNA ENST00000471220, ENST00000607042, ENST00000478738, MALAT1 were 0.689 (95%CI: 0.625–0.753), 0.958 (95%CI: 0.936–0.980), 0.714 (95%CI: 0.653–0.776) and 0.580 (95%CI: 0.510–0.650) (Fig. 9) which shows a good predictive value of lncRNA ENST00000607042 in IA patients.

**Table 3:** Logistic regression analyses of lncRNAs for predicting IA risk

Factors	B	SE	Wald	P	OR	95%CI
lncRNA ENST00000471220	−0.504	0.115	19.371	< 0.001	0.604	0.482–0.756
lncRNA ENST00000607042	−2.568	0.374	47.093	< 0.001	0.077	0.037–0.160
lncRNA ENST00000478738	−0.484	0.110	19.426	< 0.001	0.616	0.497–0.764
lncRNA MALAT1	−0.283	0.119	5.675	0.017	0.754	0.597–0.951
lncRNA ENST00000576153	0.118	0.132	0.795	0.373	1.125	0.868–1.458

Data are adjusted for age, gender, marital status, smoking, tea drinking, and physical exercise

### Stratified analysis of lncRNA ENST00000607042

Stratification analysis by various influencing factors revealed the statistically significant associations between lncRNA ENST00000607042 and IA risks in young and old individuals, males and females, smokers and non-smokers, alcohol drinkers and non-alcohol drinkers, high-salt diets and non-high-salt diets, tea drinkers and non-tea drinkers, physical exercise and no physical exercise (Table 4). These results show that lncRNA ENST00000607042 exhibit significant differences in various populations.

**Table 4:** Stratified analysis of lncRNA ENST00000607042

Stratified variables		N	ENST00000607042		*P* Value
			Controls	Cases	
Gender	Male	59/59	0.065 ± 1.061	− 2.462 ± 0.861	< 0.001
	Female	71/71	−0.054 ± 1.117	−2.397 ± 1.128	< 0.001
Age	<60	105/105	−0.075 ± 1.090	−2.423 ± 1.029	< 0.001
	≥60	25/25	0.315 ± 1.047	−2.441 ± 0.978	< 0.001
Smoking	No	123/88	−0.059 ± 1.045	−2.399 ± 1.117	< 0.001
	Yes	7/42	1.031 ± 1.416	−2.482 ± 0.769	< 0.001
Alcohol drinking	No	70/85	0.086 ± 1.089	−2.330 ± 1.035	< 0.001
	Yes	60/45	−0.101 ± 1.090	−2.608 ± 0.963	< 0.001
High-salt diets	No	107/76	−0.118 ± 1.027	−2.473 ± 1.064	< 0.001
	Yes	23/54	0.546 ± 1.221	−2.361 ± 0.949	< 0.001
Tea drinking	No	21/64	−0.056 ± 0.989	−2.462 ± 1.032	< 0.001
	Yes	109/66	0.011 ± 1.111	−2.392 ± 1.006	< 0.001
Physical exercise	No	32/75	0.100 ± 1.061	−2.533 ± 1.057	< 0.001
	Yes	98/55	−0.033 ± 1.100	−2.280 ± 0.946	< 0.001

## Discussion

Several studies have shown that dysregulation of lncRNA expression Related to a variety of diseases such as cancer, diabetes, cardiovascular and cerebrovascular diseases [29–32]. We found many differentially expressed lncRNAs and mRNAs by sequencing. GO, KEGG pathway analyses and a CNC network were constructed to elucidate the functions and possible mechanisms of differentially expressed genes. In addition, the lncRNA ENST00000607042 was found to have superior diagnostic value of IA patients.

Notably, we found that lncRNAs could be involved in immune/inflammatory response, cell adhesion and extracellular matrix by bioinformatics analyses, which we considered crucial to the pathogenesis of IA. The possible explanations for the results might be that: for the inflammatory response, studies have demonstrated that lncRNAs are key factors in inflammation-related diseases, including IA [33–36]. Recent studies have shown that the accumulation of lipids in aneurysms wall is related to the remodeling and destruction of aneurysm wall. The destruction of the aneurysm wall is related to Hemoxygenaze-1 secreted by certain inflammatory cells [37, 38]. For cell adhesion and extracellular matrix, one of the important biological processes that have been proven to be the pathogenesis of IA [39]. Extracellular matrix (ECM) serves important functions to cell adhesion, several cell adhesion molecules, such as Versican (VCAN) and Polyclonal Antibody to Integrin Alpha 10 (ITGA10), play a role in maintaining the function of ECM. The remodeling of ECM contributes significantly to the structure and integrity of intracranial arteries [40].

Most lncRNA functions are still poorly understood [41]. Therefore, the lncRNA-mRNA co-expression network was constructed based on 5 candidate lncRNAs and differentially expressed mRNA from sequencing. There are many lncRNAs-mRNAs pairs in the co-expression network that may be involved in the mechanism of IA, such as lncRNA ENST00000471220-EEF2K, lncRNA ENST00000607042-SCML4, lncRNA ENST00000576153-MMP19, lncRNA ENST00000471220-LEF1. The further GO and KEGG enrichment analyses indicated that the genes were most enriched in the T cell receptor signaling pathway, cytokines-cytokines receptor interaction and chemokine signaling pathway. Inflammation is driven by a network comprising cytokines, chemokines, their target receptors and leukocytes [42], indicating that the selected lncRNAs were closely related to the IA development. Because the inflammatory response plays an important role in IA [43].

Five candidate lncRNAs were finally selected and validated in 130 IA patients and 130 controls by qRT-PCR assay. The expression level of lncRNA ENST00000607042, lncRNA ENST00000471220, lncRNA ENST00000478738 and lncRNA MALAT1 in IA patient were lower than that of the controls (all *P* < 0.05). While the expression level of lncRNA ENST00000476153 was higher than the controls. It is worth noting that the expression levels of lncRNA ENST00000607042, lncRNA ENST00000471220, lncRNA ENST00000478738 in peripheral blood leukocytes are contradictory to sequencing. We consider it for the following reasons: (1) this may be related to the heterogeneous expression of lncRNA and the chemotaxis of central inflammation [44]. (2) The sample size used for sequencing is relatively small, and the result of false positives is inevitable. Further functional experiments are needed to confirm this speculation.

Multivariate regression analysis revealed that low expression of lncRNA ENST00000471220, lncRNA ENST00000607042, lncRNA ENST00000478738 and lncRNA MALAT1 were the risk factor of IA. The further AUC curve of lncRNA ENST00000607042 shows superior predictive value for IA, which indicated that lncRNA ENST00000607042 might serve as a biomarker for IA diagnosis and treatment. Further stratified analysis results show that lncRNA ENST00000607042 exhibit significant differences in various populations. Especially, We focus on the co-expression relationship pairs associated with lncRNA ENST00000607042 to explore the potential biological functions in IA. We found that SCML4 is associated with endothelial dysfunction and vascular remodeling [21]. CXCR4, another mRNA co-expressed with lncRNA ENST00000607042, has been reported to be likely to regulate chemotaxis and adhesion through the CXCR4–SDF-1 pathway, which is closely related to cardiac and macrovascular development [45]. All these results suggested that lncRNA ENST00000607042 may be involved in the development of IA.

Recent literature also reported the relationship between lncRNAs and IA through microarray analysis [14]. We all found many different lncRNAs, and functional analysis suggested that lncRNAs were involved mainly in regulating immune/inflammatory processes/pathways. Compared with this report, our main advantages are listed as follows. First, in addition to predicting the function of lncRNA based on the results of bioinformatics, a large sample qRT-PCR verification was conducted to ensure the reliability of the screening results; moreover, we also included other adjust common risk factors to determine further the lncRNA that may be related to IA, and draw the ROC curve to explore the value of lncRNA in predicting IA, the research content is more abundant.

There were some limitations to the current study. Firstly, the sample size used for sequencing is relatively small because it is difficult to obtain IA tissues. Secondly, The results of this study are mostly based on bioinformatics predictions, and further experiments are needed to clarify the specific role in IA. Thirdly, although we discovered good predictive value of lncRNA ENST00000607042 and related inflammatory response in IA such as chemotaxis and adhesion, The detailed mechanism of lncRNA ENST00000607042 in the development and progression of IA remains to be further explored in our future work.

## Conclusions

In summary, our study facilitates comprehensive understanding of lncRNA expression profiles in IA and reveals the close relationship between inflammatory response and IA. Moreover, the low expression of lncRNA ENST00000471220, lncRNA ENST00000607042, lncRNA ENST00000478738 and lncRNA MALAT1 were independent risk factors for IA. LncRNA ENST00000607042 has superior diagnostic value which might be served as novel biomarkers for IA risk.

## Supplementary information

**Additional file 1: Table S1.** Clinical characteristics of included patients.

**Additional file 2: Table S2.** List of the primers used for qRT-PCR experiments.

## Data Availability

All the relevant data and Additional file 2 are all available. The datasets generated and analysed during the current study are available in the [GEO: GSE158558] repository, [http://www.ncbi.nlm.nih.gov/geo/]”.

## Abbreviations

IA: Intracranial aneurysm
LncRNA: Long non-coding RNA
mRNA: Messenger RNA
STAs: Superficial temporal arteries
GO: Gene Ontology
KEGG: Kyoto Encyclopedia of Genes and Genomes
CNC: Coding non-coding
qRT-PCR: Quantitative real-time polymerase chain reaction
ROC curve: Receiver operating characteristic curve
aSAH: Aneurysmal subarachnoid hemorrhage
SMCs: vascular smooth muscle cells
BP: Biological processes
CC: Cellular components
MF: Molecular functions
EEF2K: Eukaryotic elongation factor 2 kinase
SCML4: Scm polycomb group protein-like 4
MMP19: Matrix metalloproteinases 19
LEF1: Lymphoid enhancer factor 1
