# Supplementary material for: Exploring the association of long noncoding RNA expression profiles with intracranial aneurysms, based on sequencing and related bioinformatics analysis
Source: BMC Med Genomics. 2020 Oct 6;13:147. doi: 10.1186/s12920-020-00805-x (PMC7542138; doi:10.1186/s12920-020-00805-x)
Supplement: Supplementary file 1 — Additional file 1: Table S1. Clinical characteristics of included patients. [file 12920_2020_805_MOESM1_ESM.doc]

**Table S1 Clinical characteristics of included patients**

| Characteristics | IA sample (*n=*4) | STA sample (*n=*4) | T/χ^2^ | *P* Value |
| --- | --- | --- | --- | --- |
| Age | 57.50±3.00 | 56.75±2.75 | -0.368 | 0.725 |
| Gender |  |  | 0 | 1.000* |
| Male | 1 (25) | 1 (25) |  |  |
| Female | 3 (75) | 3 (75) |  |  |

* Fisher 's exact test.
