# Supplementary material for: Exploring the association of long noncoding RNA expression profiles with intracranial aneurysms, based on sequencing and related bioinformatics analysis
Source: BMC Med Genomics. 2020 Oct 6;13:147. doi: 10.1186/s12920-020-00805-x (PMC7542138; doi:10.1186/s12920-020-00805-x)
Supplement: Supplementary file 2 — Additional file 2: Table S2. List of the primers used for qRT-PCR experiments. [file 12920_2020_805_MOESM2_ESM.docx]

| **Table S2** List of the primers used for qRT-PCR experiments | | |
| --- | --- | --- |
| Name | Primer | Sequence（5’-3’） |
| β-actin | Forward | GTGGCCGAGGACTTTGATTG |
|  | Reverse | CCTGTAACAACGCATCTCATATT |
| ENST00000508090 | Forward | AGGAGTTTCAGGAGGGTATTG |
|  | Reverse | CAGGAGGCAGATTTCTGTGTTA |
| ENST00000576153 | Forward | ACTCACTCTTCCCTCTGTTGG |
|  | Reverse | GGCTGGTAGGAAGTAAATGC |
| ENST00000471220 | Forward | GTGTCTGCTTATGTCGGTGAG |
|  | Reverse | TTCCTATGATCTTTCTGTGCC |
| ENST00000478738 | Forward | CTGTTAGGCAGCAACAGTGC |
|  | Reverse | CCCAGATTGGCACCTCGAAA |
| ENST00000607042 | Forward | CGTGGTAAACGTAAACTTACTATTG |
|  | Reverse | CCGCAAGAACTTCATGCTATA |
| MALAT1 | Forward | GCTCTGTGGTGTGGGATTGA |
|  | Reverse | GTGGCAAAATGGCGGACTTT |
| ENST00000579688 | Forward | ACCCCAGGAAATCAGTGTCCA |
|  | Reverse | CTCAAGCGCAGCCTTTTCTCC |
